# Altered gut microbiomes are associated with the symptomatic status of unruptured intracranial aneurysms

**DOI:** 10.3389/fnins.2022.1056785

**Published:** 2022-12-22

**Authors:** Kaijian Sun, Ying Cao, Yiting Chen, Qing Peng, Yugu Xie, Yunhao Luo, Hao Tian, Xin Li, Meiqin Zeng, Xin Zhang, Xifeng Li, Shixing Su, Xuying He, Chuanzhi Duan, Haitao Sun

**Affiliations:** ^1^The National Key Clinical Specialty, Guangdong Provincial Key Laboratory on Brain Function Repair and Regeneration, Department of Cerebrovascular Surgery, Engineering Technology Research Center of Education Ministry of China on Diagnosis and Treatment of Cerebrovascular Disease, Neurosurgery Center, Zhujiang Hospital, The Neurosurgery Institute of Guangdong Province, Southern Medical University, Guangzhou, Guangdong, China; ^2^Department of Laboratory Medicine, Clinical Biobank Centre, Microbiome Medicine Center, Zhujiang Hospital, Southern Medical University, Guangzhou, Guangdong, China; ^3^Key Laboratory of Mental Health of the Ministry of Education, Guangdong–Hong Kong–Macao Greater Bay Area Centre for Brain Science and Brain-Inspired Intelligence, Southern Medical University, Guangzhou, Guangdong, China

**Keywords:** symptomatic intracranial aneurysms, gut microbiome, gut-brain axis, 16s rDNA sequencing, 16S rDNA sequence

## Abstract

**Background:**

Gut microbiome has recently been recognized as an important environmental factor affecting the occurrence and development of unruptured intracranial aneurysms (UIA). This study aimed to investigate the relationship between gut microbiome and symptomatic UIA, which is a predictor of instability and a high propensity to rupture.

**Methods:**

A total of 132 patients including 86 asymptomatic UIA and 46 symptomatic UIA were recruited in the study. The composition of gut bacterial communities was determined by 16S ribosomal RNA gene sequencing. In addition, Phylogenetic Investigation of Communities by Reconstruction of Unobserved States (PICRUSt) was used to predict the functional composition of the gut microbiome.

**Results:**

There is no difference in the fecal microbial alpha diversity between symptomatic and asymptomatic UIA, but gut microbiome composition changed significantly. At the order level, the relative abundance of *Clostridiales* was significantly enriched in the symptomatic compared with asymptomatic UIA (*p* = 0.043). In addition, similar alterations were observed at the family levels of *Ruminococcaceae*. The Linear discriminant analysis (LEfSe) revealed *Fournierella*, *Ruthenibacterium*, and *Anaerotruncus* as discriminative features in the symptomatic group. Notably, functional differences in gut microbiome of patients with symptomatic UIA included decreased propionate metabolism pathway and enrichment of peptidoglycan biosynthesis pathways.

**Conclusion:**

The present study comprehensively characterizes gut microbiome in a large cohort of different risk statuses of UIA patients and demonstrates the potential biological function of gut microbiome involved in the development of UIA. It may provide additional benefits in guiding UIA management and improving patient outcomes.

## Introduction

Unruptured intracranial aneurysms (UIA) affect 7% of the Chinese adult population and are the main cause of subarachnoid hemorrhage with high case fatality and morbidity (Li et al., 2013). Preventive endovascular or neurosurgical aneurysm treatment options often carry a substantial risk of procedure-related complications. Hence, it warrants adequate risk assessment to identify unstable UIA timely and precisely. However, the current commonly used prediction model as the PHASES score is inadequate for discriminating UIA in high rupture risk. A low PHASES score does not imply a negligible likelihood of aneurysm rupture, and the classifier has a low sensitivity (Bijlenga et al., 2017). In addition, up to 70% of ruptured IAs are smaller than 7 mm. To date, risk assessment of intracranial aneurysms remains a formidable clinical challenge. Therefore, it is essential to identify novel indicators that may improve the current risk assessment of UIA.

Although most UIA patients are asymptomatic, the following typical symptoms strongly suggest an unstable state and a high rupture risk of UIA, such as sentinel headache (SH) characterized by severe and acute onset headache (Nakagawa et al., 2018), and oculomotor nerve palsy (ONP) caused by local mass effect (Guresir et al., 2011). Growing evidence suggested that the gut microbiome is a pivotal environmental factor affecting host metabolism and immune homeostasis. A recent study showed that antibiotic depletion of the gut microbiome reduced the incidence of intracranial aneurysms in rats, and the mechanism was associated with reduced macrophage infiltration and levels of inflammatory cytokines (Shikata et al., 2019). Further research showed that patients with UIA could be distinguished from healthy controls based on 47 differentially enriched species (Li et al., 2020). Recently a Japanese study found that *Campylobacter* may be associated with the rupture of cerebral aneurysms (Kawabata et al., 2022). However, the stress response following subarachnoid hemorrhage can have a dramatic disturbance on the gut microbiome, making it difficult to serve as a tool for rupture risk assessment. And so far, the relationship between gut microbiome and unstable UIA is still unclear. Therefore, our study aimed to investigate the association between gut microbiome and symptomatic UIA in a Chinese cohort to find out the dominant gut microbiome in symptomatic UIA.

## Materials and methods

### Studying population

The study was conducted in accordance with the guidelines of the Declaration of Helsinki and was approved by the Ethics Committee of Zhujiang Hospital, Southern Medical University before initiating a cross-sectional study (reference number: 2021-KY-038-02). And the informed consent was obtained from all patients. Patients diagnosed with UIA at Zhujiang Hospital were enrolled from September 2020 to December 2021.

Inclusion criteria were as follows: Patients diagnosed with UIA through digital subtraction angiography (DSA), asymptomatic defined as incidental detection on physical examination or symptomatic patients with UIA have clinical signs including headache (development of a sudden and severe headache on the ipsilateral side of the aneurysms within 2 weeks of admission without prior history of headache within the previous 5 years; (Fu et al., 2021) or oculomotor nerve palsy (with one or several symptoms of sudden unilateral loss of visual acuity, poor pupil response to light, ptosis, and extraocular muscle palsy) (Guresir et al., 2011).

The exclusion criteria were as follows: (1) A ruptured aneurysm has occurred; (2) Family history of intracranial aneurysm, persistent infectious disease, inflammatory bowel disease, irritable bowel syndrome, autoimmune disease, liver disease, kidney disease, cancer, and diarrhea, and use of antibiotics or probiotics 2 months before specimen collection; (3) fusiform or dissecting aneurysm; (4) previously treated aneurysm; (5) Aneurysms other than intracranial aneurysms; and (6) The diagnostic images or clinical data of DSA without IA were incomplete.

### Clinical data collection

Demographic and clinical data, which include age, gender, smoking history, hypertension, cardiac disease, hyperlipidemia, stroke history, and history of diabetes were recorded by the researcher after they were admitted to the hospital. Aneurysm characteristics, size and location were collected from the image of DSA.

### Fecal samples collection

Each participant was asked to use a sterile container to collect fresh fecal samples once they were admitted to the hospital. All fecal samples within half an hour were dispensed in 2 ml Eppendorf tubes (Hamburg, Germany), each tube packing 180 ± 20 mg, immediately placed in −80°C until analysis. Ensuring that samples are as fresh and pollution-free as possible.

### Bacterial DNA extraction and 16rRNA gene sequencing

Fecal DNA extraction. Fecal bacterial DNA was extracted using Magnetic Soil and Stool DNA Kit (TIANGEN BIOTECH, China) according to the manufacturer’s instructions, and stored at −20°C prior until further analysis. The quantity and quality of extracted DNA was measured using the NanoDrop ND-1,000 spectrophotometer and agarose gel electrophoresis.

16S rRNA Amplicon sequencing. PCR amplification of the bacterial 16S rRNA gene V3–V4 regions was performed using the forward primer 341F (5′–CCTAYGGGRBGCASCAG–3′) and the reverse primer 806R (5′–GGACTACNNGGGTATCTAAT–3′). Sample-specific 6-bp barcodes were incorporated into the primers for multiplex sequencing. All PCR reactions were carried out with 15 μL of Phusion^®^ High-Fidelity PCR Master Mix (New England Biolabs, Ipswich, MA, United States), 0.2 μM of forward and reverse primers, and about 10 ng template DNA. Thermal cycling consisted of initial denaturation at 98°C for 1 min, followed by 30 cycles of denaturation at 98°C for 10 s, annealing at 50°C for 30 s, elongation at 72°C for 30 s, and finally 72°C for 5 min. PCR amplicons were purified with Qiagen Gel Extraction Kit (Qiagen, Germany) by agarose gel electrophoresis (2%). Sequencing libraries were generated using TruSeq^®^ DNA PCR-Free Sample Preparation Kit (Illumina, USA) following the manufacturer’s recommendations. The library quality was assessed on the Qubit@2.0 Fluorometer (Thermo Scientific, Carlsbad, CA, United States) and Agilent Bioanalyzer 2,100 system. At last, the library was sequenced on an Illumina NovaSeq platform (San Diego, CA, United States).

### Bioinformatic analysis of 16S rRNA gene sequencing

The procedures for total fecal DNA extraction, amplification, and sequencing of the V3–V4 region of the 16S rRNA genes as well as the construction of the sequencing library were illustrated in previous publications. The downstream amplicon bioinformatic analyses were performed with EasyAmplicon v1.12 (Liu et al., 2021). Dereplication was performed using the deep full length command of VSEARCH (v2.15.2 2.0.3). Then, the non-redundant sequences were denoised into amplicon sequence variants (ASVs) *via* the -unoise3 command of USEARCH (v10.0.240). The feature (ASV) table was created with VSEARCH–USEARCH global. Taxonomic classification of ASVs was achieved using the syntax algorithm of USEARCH based on the Ribosomal Database Project (RDP) training set v16. The sequences of all samples were rarefied to 30,000 for the downstream diversity analysis. Alpha diversity analysis was carried out using the vegan package (v2.5-6) in R v4.1.0. Differences in Richness index, Shannon’s index and the ACE index between groups were evaluated by Wilcoxon test analysis. The weighted bray curtis and unifrac distance matrix were generated using USEARCH- beta_div. Beta diversity calculations were performed by principal coordinate analysis (PCoA). Permutational multivariate analysis of variance was used to define the significance of differences between groups. The R package ggplot2 was used to visualize the results of the diversity analysis. The Venn diagram illustrating ASV overlapping between groups was generated using the Venn Diagram package. The compositions of the microbial community in two groups were presented as stacked bar plots at the phylum and family levels, respectively. The edgeR package was applied to evaluate the differences in ASV abundance in two groups, and the Benjamini–Hochberg method was used to control the false discovery rate (FDR). The differences in bacterial abundances at different bacterial classification levels were analyzed by linear discriminant analysis (LDA) of effect size (LEfSe). Pathway enrichment analysis was performed utilizing the database from the Kyoto Encyclopedia of Genes and Genomes (KEGG) with PICRUSt2 (Douglas et al., 2020).

### Statistical analysis

SPSS (IBM Corp., SPSS 23.0, Armonk, NY, United Status) was used for data analysis. The normally distributed continuous variables between two groups were presented with means with standard deviation (mean ± SD) and analyzed by unpaired Student’s *t*-test, while non-normally distributed were analyzed by the Wilcoxon rank test. Categorical variables were compared by the χ^2^ test. The abundance of specific bacteria between groups was analyzed with Welch’s *t*-test corrected with Bonferroni. *P* < 0.05 (all two-sided calculated) was regarded as significant. Spearman correlation analysis was used to determine the correlation between gut microbiota and clinical indicators. The results were corrected by FDR. FDR <0.05 was considered significant.

## Results

### Sample demographic data

A total of 132 subjects with DSA diagnosis of UIA were recruited from September 2020 to December 2021. Among them, 43 UIA patients showed typical symptoms and 86 were asymptomatic. Typical symptoms included sentinel headache (*n* = 40) and ONP (*n* = 6), of note, three patients presented both symptoms with headache and ONP (Supplementary Figure 1). Characteristics of the asymptomatic UIA and symptomatic UIA patients are shown in Table 1. The patients were similarly distributed in terms of age, gender, and tobacco user. There were no differences between the two groups regarding comorbidities such as cardiac disease (myocardial infarction and angina), hyperlipemia, and stroke history. No difference was observed in the location of UIA (internal carotid artery; middle cerebral artery; anterior communicating artery or posterior communicating artery). Whereas, the rate of multiple aneurysms was significantly higher in symptomatic UIA patients (30.4 vs. 12.8%, *p* = 0.014).

**TABLE 1:** Demographic and clinical features of the study cohort.

	Asymptomatic intracranial aneurysm (*n* = 86)	Symptomatic intracranial aneurysm (*n* = 46)	*P*-value for trend
**Patient characteristics**			
Age, years*	55.9 ± 10.6	55.8 ± 12.1	0.349
Gender (female)^§^	58/86 (67.4%)	36/46 (78.3%)	0.191
Tobacco use^§^	6/86 (7.0%)	5/46 (10.9%)	0.395
**Comorbidities^§^**			
Hypertension^§^	32/86 (37.2%)	19/46 (41.3%)	0.645
Cardiac disease^§^	2/86 (2.3%)	0	0.297
Hypercholesterolemia^§^	3/86 (3.5%)	2/46 (4.3%)	0.805
Stroke history^§^	5/86 (5.8%)	0	0.095
Diabetes mellitus^§^	9/86 (10.5%)	3/46 (6.5%)	0.453
**Aneurysm characteristics^§^**			
Multiplicity^§^	11/86 (12.8%)	14/46 (30.4%)	0.014
Irregular shape^§^	10/86 (11.6%)	8/46 (17.4%)	0.358
**Size**			
Average diameter*, mm	5.5 ± 3.9	6.0 ± 3.8	0.574
**Location^§^**			0.571
ICA	36/86 (41.9%)	23/46 (50.0%)	
MCA	6/86 (7.0%)	9/46 (19.6%)	
AComA	8/86 (9.3%)	4/46 (8.7%)	
PComA	4/86 (4.7%)	2/46 (4.3%)	
Posterior	4/86 (4.7%)	2/46 (4.3%)	

*Mean ± SD, ^§^*n* (%), For the difference comparison of clinical characteristics between the groups, the Student’s *t*-test was employed in cases of continuous normally distributed data. Categorical variables were compared by the χ^2^ test. Hypertension is defined as blood pressure >140/90 mmHg. Cardiac disease is defined as the history of myocardial infarction or angina. ICA, internal carotid artery; MCA, middle cerebral artery; AComA, anterior communicating artery; PComA, posterior communicating artery.

### Alterations of overall microbial composition in symptomatic unruptured intracranial aneurysms patients

**FIGURE 1:**
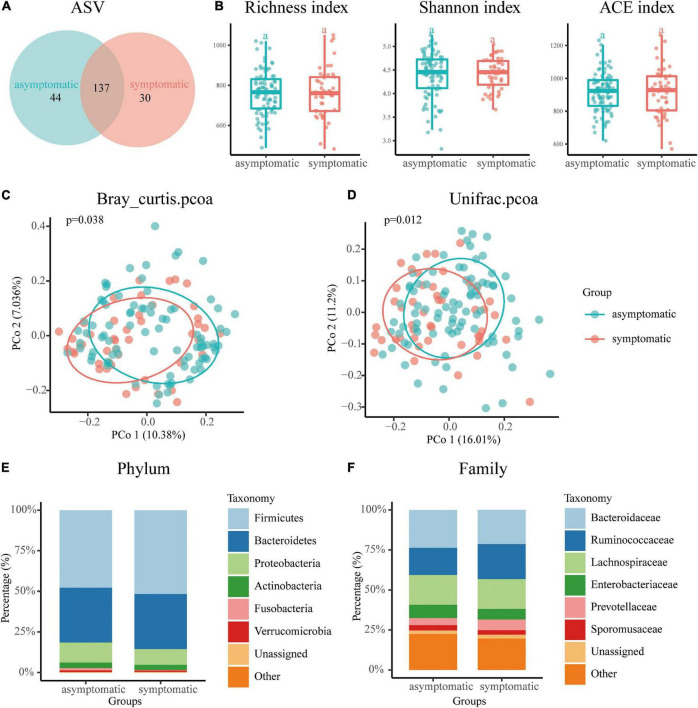
Overview of the gut microbiome in the asymptomatic UIA and symptomatic UIA groups. **(A)** The Venn diagram indicated the existence of ASVs in each group. **(B)** The Richness index, Shannon index, and ACE richness estimator were used to determine the microbiome richness, and no significant differences were observed between the asymptomatic UIA and symptomatic UIA groups. The horizontal bar within each box represents the median location, the box represents the interquartile range and the whiskers end indicates the minimum and maximum values. **(C,D)** Principal coordinate analysis illustrated the grouping patterns of the asymptomatic UIA and symptomatic UIA groups using both weighted Bray Curtis and UniFrac distances. **(E)** Dominant phyla in each group. The main gut microbiome were *Firmicutes*, *Bacteroidetes*, *Proteobacteria*, *Actinobacteria*, and *Verrucomicrobia* at the phylum level. **(F)** Dominant family in each group. The main gut mocrobiome were *Bacteroidaccae*, *Ruminococcacea*, *Lachnospiraceae*, *Enterobacteriacea*, *Prevotellaceae*, and *Sporomusaceae* at the family level.

As shown in Figure 1A, Venn diagram displayed 44 unique ASVs in the asymptomatic group and 30 unique ASVs in the symptomatic group. A total of 137 ASVs were shared by both groups (Figure 1A). For Alpha diversity index, we use the Richness index, Shannon index and ACE richness estimator to evaluate species richness and no significant difference was observed in the above indicators between the symptomatic and asymptomatic UIA groups (Figure 1B). To assess variation in microbial composition, we performed principal co-ordinates analysis based on Bray curtis dissimilarity and weighted UniFrac distances. The β-diversity analysis demonstrated significant differences in gut taxonomic composition between symptomatic and asymptomatic UIA patients by both two methods (*p* = 0.038, *p* = 0.012, Figure 1C). Then, the composition of the gut microbiome at the phylum and family levels in two groups was evaluated by stacking column diagram. The predominant phyla were *Firmicutes*, *Bacteroidetes*, *Proteobacteria*, and *Actinobacteria* (Figure 1D). At the family level, *Bacteroidaceae*, *Ruminococcaceae*, *Lachnospiraceae*, *Enterobacteriaceae*, *Prevotellaceae*, and *Sporomusaceae* were the most abundant (Figure 1F). Among them, *Firmicutes*, *Prevotellaceae*, and *Ruminococcaceae* tend to increase in symptomatic UIA.

We performed a spearman correlation analysis of gut microbiota with clinical indicators of IA, and the results were corrected by FDR. The results showed that *Lawsonibacter* was positively correlated with aneurysm size (*p* < 0.05), while *Ruminiclostridium* and *Burkholderia* were negatively correlated with aneurysm max diameter (*p* < 0.01) (Supplementary Figure 2).

### Differentially abundant gut microbiome in symptomatic versus asymptomatic unruptured intracranial aneurysms

**FIGURE 2:**
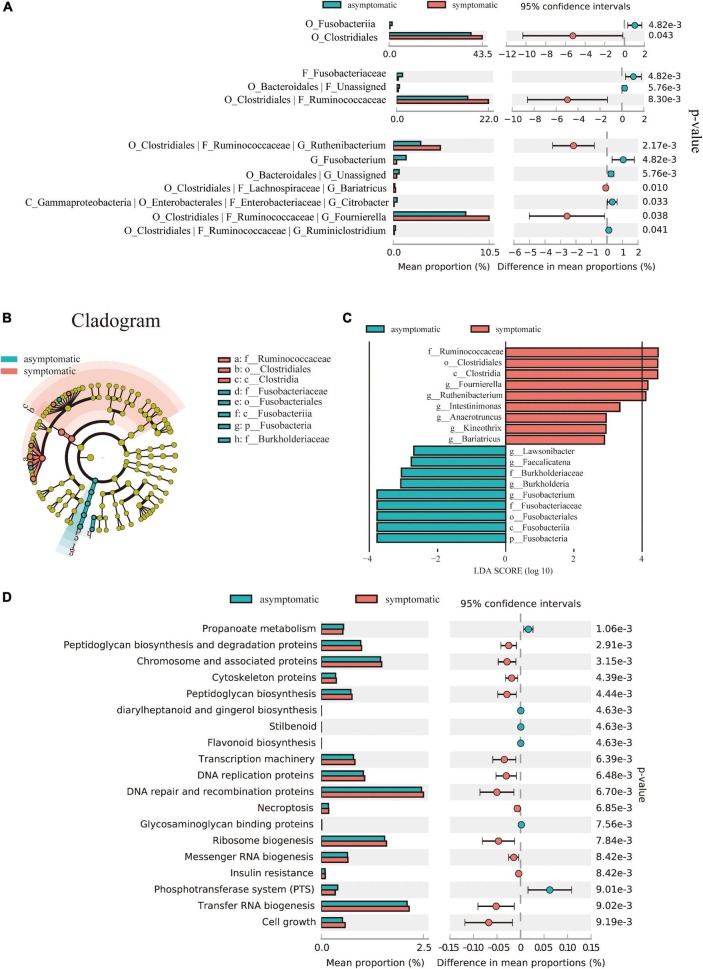
Alterations in the composition and function of the gut microbiome. **(A)** Comparison of the representative taxonomic abundance between symptomatic and asymptomatic UIA groups. Welch’s *t*-test indicated the significant enrichment of *Clostridiales* in symptomatic group and also in its corresponding family (*Ruminococcaceae*) and genus. **(B)** The cladogram showed taxonomy differences between the symptomatic and asymptomatic UIA groups according to Linear discriminant analysis effect size (LEfSe). **(C)** LEfSe analysis revealed discriminative taxa between symptomatic and asymptomatic UIA. **(D)** Functional comparison of the gut microbiome between the asymptomatic and symptomatic UIA was analyzed with the KEGG pathway.

To further identify differentially abundant taxa, we performed Welch’s *t*-test and LEfSe analysis on the fecal microbiome composition in symptomatic vs. asymptomatic UIA. However, no significant changes were observed at the phylum level between the two groups. At the genus level, there were seven bacterial taxa showing significantly different relative abundances between the two groups (Figure 2A). Interestingly, most of the bacteria enriched in the symptomatic group belong to the *Ruminococcaceae* family, *Clostridiales* order. Consistently, the linear discriminant analysis (LDA) distribution diagram analysis (LDA score > 2.0, *p* < 0.05) showed a clear alteration of the microbiome characterized by higher *Ruminococcaceae, Fournierella*, *Ruthenibacterium*, and *Anaerotruncus* levels in symptomatic UIA patients. However, *Fusobacteria* levels were significantly decreased in symptomatic patients (Figures 2B,C).

### Predicted microbiological function analysis of microbiome in symptomatic and asymptomatic unruptured intracranial aneurysms patients

To further reveal the potential connections between gut microbiome and unstable UIA, KEGG functional orthologs were predicted with PICRUSt2. We observed significant differences between patients at different UIA stages and indicated that symptomatic UIAs may involve a universal metabolic disturbance: The symptomatic UIA group had 19 functional orthologs changed when compared with asymptomatic UIA patients. Notably, propanoate metabolism was significantly decreased in gut microbiome of symptomatic UIA group (*p*-value = 0.0011) while peptidoglycan biosynthesis pathways were significantly enriched (*p*-value = 0.003) (Figure 2D). Moreover, enrichment analysis revealed significant upregulation of genetic information processing in symptomatic UIAs groups like the expression of chromosome associated protein (correlated *p*-value = 3.15 × 10^–3^), transcription machinery (correlated *p*-value = 6.39 × 10^–3^) DNA replication proteins (correlated *p*-value = 6.48 × 10^–3^), ribosome biogenesis (correlated *p*-value = 7.84 × 10^–3^), and transfer RNA biogenesis (correlated *p*-value = 9.02 × 10^–3^) compared to asymptomatic UIAs. Expression levels of insulin resistance may be positively associated with the risk of severe symptoms, and this was also supported by epidemiological studies of coronary artery disease (Mahmood et al., 2014). Besides, the gut microbiome of asymptomatic UIA patients had enriched another six functional orthologs including propanoate metabolism (correlated *p*-value = 1.06 × 10^–3^), diarylheptanoid and gingerol biosynthesis (correlated *p*-value = 4.63 × 10^–3^), stilbenoid (correlated *p*-value = 4.63 × 10^–3^), flavonoid biosynthesis (correlated *p*-value = 4.63 × 10^–3^), glycosaminoglycan binding protein (correlated *p*-value = 7.56 × 10^–3^), and phosphotransferase system (correlated *p*-value = 9.01 × 10^–3^).

## Discussion

Growing evidence has suggested that gut microbiome is a critical environmental factor that contributes to physiology and pathology of IA (Shikata et al., 2019; Li et al., 2020; Kawabata et al., 2022). In our study, we delineated the community structure of fecal microbiome in patients with asymptomatic and symptomatic UIA through 16S rRNA gene sequencing and characterized the gut microbiome and its function associated with unstable UIA. The noteworthy finding was that the *Ruminococcaceae* family was highly enriched in symptomatic UIA patients. Besides, peptidoglycan biosynthesis and propanoate metabolism pathways were the most significantly altered functional traits. In the Lefse analysis, several kinds of gut microbiome between symptomatic and asymptomatic UIA patients were significantly different. Notably, genus of *Fournierella*, *Ruthenibacterium*, and *Anaerotruncus*, highly enriched in symptomatic UIA, were all clustered into *Ruminococcaceae* family.

A previous study found that arterial stiffness correlates negatively with the abundance of *Ruminococcaceae* family bacteria (Menni et al., 2018). Similarly, our study found that the weak wall of unstable aneurysms may be related to the increased abundance of *Ruminococcaceae*. Additionally, *Ruminococcaceae* has a higher relative abundance in the abdominal aortic aneurysm group compared to the control group in a cross-sectional investigation (Ji et al., 2021), and a genus from *Ruminococcaceae* was demonstrated to be positively correlated with the aneurysm’s diameter (Xie et al., 2020). A study believed *Ruminococcaceae* may increase the incidence of stroke risk and *Ruminococcaceae* was higher in stroke patients with poorer outcomes. The exact mechanical explanations deserve exploring and it is inevitable that a significant dysbiosis of microbiome composition in stroke patients (Chang et al., 2021). A randomized controlled trial found that *Fournierella* and several genera from the *Ruminococcaceae* family were significantly associated with intrahepatic fat and could be affected by lifestyle intervention (Yaskolka Meir et al., 2021). It is reported that the relative abundance of *Anaerotruncus* increases when there is gut dysbiosis, which may have a deleterious impact on health (Hwang et al., 2017). Bailén et al. (2020) observed that healthy patients who were on a higher intake of saturated fatty acids dietary had more abundant *Anaerotruncus* genus, which is associated with obesity. Elevated lipid infiltration has been shown to be closely associated with aneurysm rupture. Our study may explain aneurysm lipid infiltration partly from the elevated abundances of *Fournierella* and *Anaerotruncus*. Their potential as intervention targets for intracranial aneurysms should be explored in follow-up studies.

It has been proposed that sentinel headache is caused by microbleeds or warning leaks before rupture, such as tearing in UIA. Oculomotor nerve palsy may result from the rapid growth or wall structural changes of UIA (Fu et al., 2021). A multidisciplinary consultation was conducted to evaluate and determine these symptoms in clinical scenarios. In terms of gut microbiome, the metagenomic study by Li et al. (2020) also found that the *Ruminococcus* and *Fusobacterium* were significantly elevated in the unruptured aneurysm group compared with the control group but did not differentiate the rupture risk of these unruptured aneurysms. Recently, Kawabata et al. (2022) reported that alterations in gut microbiome composition were associated with aneurysmal subarachnoid hemorrhage. However, the differential microbiome shown in their study showed no difference between the symptomatic and asymptomatic UIA groups of the current study, which imply the complexity of the pathological mechanism of IA and such microbiome-based models cannot be extrapolated because of geographical and dietary differences (He et al., 2018). In clinical practice, the connection between neurological symptoms and aneurysms is still problematic and a common cause for consultation (Dodick, 2002). A better comprehension of related headaches and ONP could contribute to IA diagnosis before their rupture and distinguish the unstable symptomatic aneurysms from the low-risk ones, since the therapy is different with an increased risk of rupture (Rinkel et al., 1998; Wermer et al., 2007). The characteristics of the microbiome of symptomatic UIA in this study may provide a deeper insight into the progression of UIA and the relation between warning symptoms and aneurysms. In our cohort, only typical symptoms like sentinel headache and oculomotor nerve palsy were investigated. The relation of gut microbiome and other symptoms related to UIA including chronic headache and (Waga et al., 1975; Samejima et al., 1990) migraine may merit in-depth investigation (Okawara, 1973).

Communication between the central nervous system and the gut microbes known as the “gut-brain axis” is quite attractive for mediators like immune-modulatory microbial metabolites. We discovered a significant decrease in propanoate metabolism in the gut microbiome of symptomatic UIA patients. Short-chain fatty acids are produced by gut bacteria and affect host immune homeostasis (Bartolomaeus et al., 2019). Propionic acid maintains immune cell function and has an immune-modulatory role in protecting from hypertensive cardiovascular damage (Bartolomaeus et al., 2019). It is acknowledged that inflammation affects the formation and growth of IAs. Decreased propionic acid synthesis capacity in the gut microbiome of patients with advanced aneurysms may reduce this protective effect, leading to increased inflammatory infiltration of the aneurysm and instability. We also found an increase in peptidoglycan biosynthesis in the unstable group which is the main component of the bacterial cell wall. Previous studies have shown that it can stimulate the production of inflammatory cytokines such as TNF-α and interleukin, activating neutrophils and lymphocytes and increasing vascular endothelial cells’ permeability (De Marzi et al., 2015). Recent studies have shown that peptidoglycan derived from the gut microbiome can be translocated into the brain and is a key mediator of gut-brain communication (Okawara, 1973; Gabanyi et al., 2022). It is established that Nod receptors in the brain sense peptidoglycan fragments, convey inflammatory signals and alter tissue function and development in the brain (De Marzi et al., 2015; Wolf and Underhill, 2018), all of which may contribute to the instability of IAs and lead to symptom aneurysms. In addition, peptidoglycan fragments are reported to be necessary for immune cell development including T cells (Martinic et al., 2017; Wolf and Underhill, 2018). Lymphocytic infiltrates in patient IA tissue specimens and have been shown to play an important role in aneurysms and related diseases such as atherosclerosis. Sawyer et al. (2016) demonstrated that the IA rupture was suppressed in mice lacking T cells, indicating the involvement of T cells in mechanisms generating the rupture of IAs. We conjecture that the elevated peptidoglycan synthesis pathways may affect the progression of IA through increased infiltration of T cells. Together, these results suggest that gut microbiome may be correlated with symptoms by regulating metabolic pathways.

Our analysis demonstrated that the rate of multiple aneurysms was significantly higher in symptomatic UIA patients (30.4 vs. 12.8%, *p* = 0.014). Several explanations can account for this. Former studies show that compared to single aneurysms, individuals with multiple aneurysms show a higher risk for IA development and instability, which may result in symptomatic UIA. A population-based retrospective cohort study by Jabbarli et al. (2018) manifested that the specific hemorheological features contribute to multiple aneurysms, which may be a share risk factor for the development of symptomatic UIA. Besides, the association between multiple and symptomatic UIA may be a matter of probability, for multiple aneurysms have a greater chance to manifest symptoms.

## Limitation

Our study has several limitations. First, the composition of the gut microbiomes is influenced by various factors such as dietary habits, obesity and gender, and evident differences are found in the microbiomes derived from different studies performed in different places, however, these factors were not investigated in your study. Second, this study was conducted in a single center, and further inclusion of multi-center, multi-regional, and multi-ethnic populations will consolidate the results of this study. Third, only typical symptoms like headache and oculomotor palsy were used for statistical analysis, other symptoms like vomiting and subarachnoid hemorrhage deserve other deep research. Finally, the causal relationship between microbiome and unstable aneurysms requires further experimental verification of fecal microbiome transplantation.

## Conclusion

The present study comprehensively characterizes gut microbiome in a large cohort of different risk statuses of UIA patients and demonstrates the potential biological function of gut microbiome involved in the development of UIA. It may provide additional benefit in guiding UIA management and improving patient outcomes.

## Data availability statement

The raw data supporting the conclusions of this article will be made available by the authors, without undue reservation.

## Ethics statement

This study was approved by the Ethics Committee of Zhujiang Hospital, Southern Medical University before initiating a cross-sectional study (reference number: 2021-KY-038-02). The patients/participants provided their written informed consent to participate in this study.

## Author contributions

HS and CD contributed to conception and design of the study. KS, YtC, YC, and QP designed experiments and prepared the manuscript. YL, XL, MZ, and XfL collected the patient samples and information. YC, YX, SS, and XH performed the statistical analysis. KS, HT, XZ, and XfL revised the manuscript. All authors contributed to manuscript revision, read and approved the submitted version.

## Supplementary material

Supplementary Figure 1 Flowchart of the process of patient selection according to the inclusion and exclusion criteria.

Supplementary Figure 2 Associations of altered gut microbes with clinical indicators. Heat map of the Spearman’s rank correlation coefficient of altered gut microbes for symptomatic UIA and IA-related clinical indicators. Red rectangle indicates positive associations between these microbial species and clinical indicators, blue rectangle indicates negative associations. The statistical significance was denoted on the rectangle (**p* < 0.05 and ^**^*p* < 0.01).
