# Supplementary figures and images for: Altered gut microbiomes are associated with the symptomatic status of unruptured intracranial aneurysms
Source: Front Neurosci. 2022 Dec 22;16:1056785. doi: 10.3389/fnins.2022.1056785 (PMC9814123; doi:10.3389/fnins.2022.1056785)

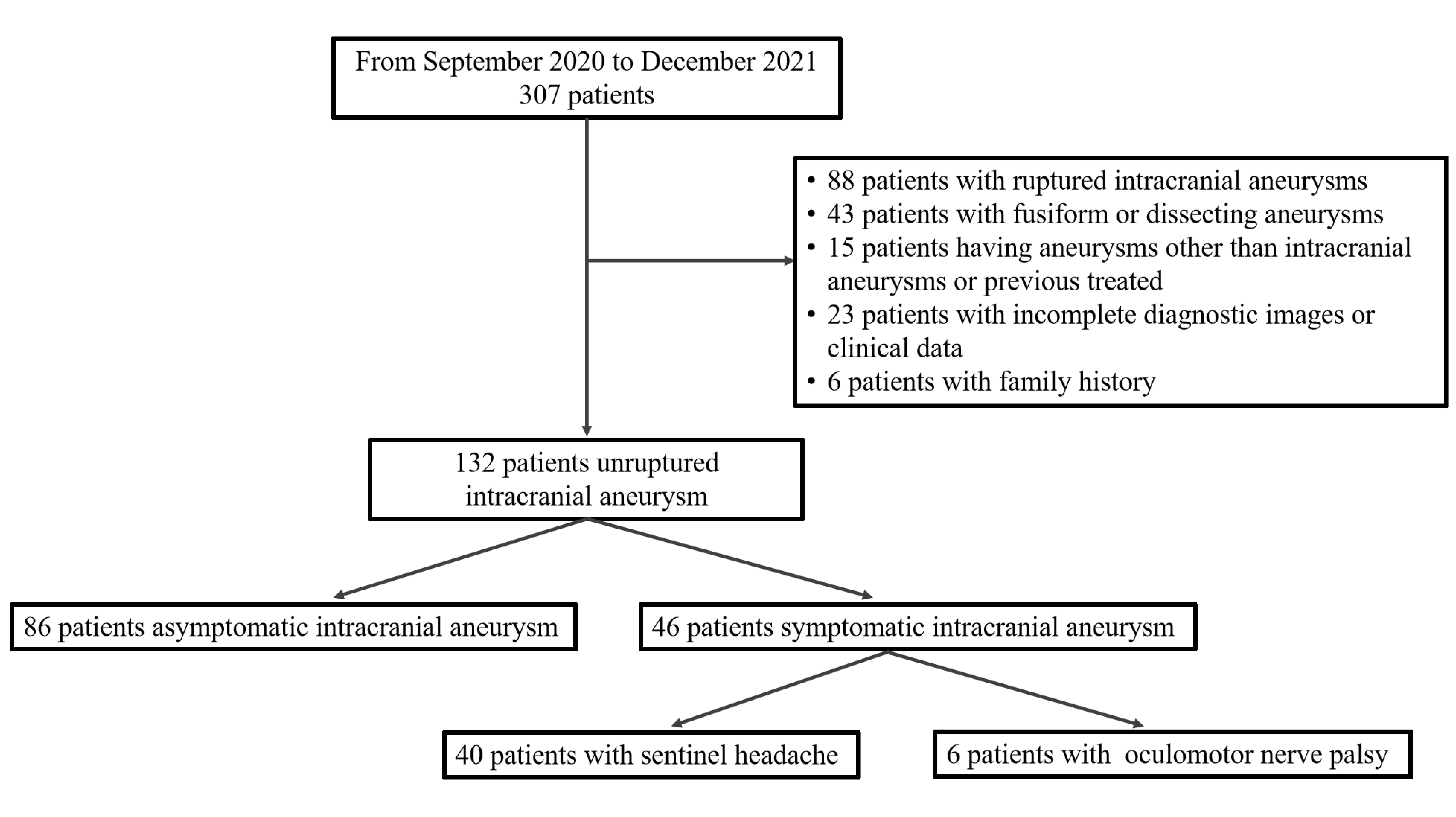

Supplement: Supplementary Figure 1 — Flowchart of the process of patient selection according to the inclusion and exclusion criteria. [file Image_1.TIF]
